# The Quest for Signals in Noise: Leveraging Experiential Variation to Identify Bilingual Phenotypes

**DOI:** 10.3390/languages6040168

**Published:** 2021-10-15

**Authors:** Anne L. Beatty-Martínez, Debra A. Titone

**Affiliations:** Department of Psychology, McGill University, Montréal, QC H3A 1G1, Canada

**Keywords:** bilingualism, language variation, individual differences, bilingual phenotypes

## Abstract

Increasing evidence suggests that bilingualism does not, in itself, result in a particular pattern of response, revealing instead a complex and multidimensional construct that is shaped by evolutionary and ecological sources of variability. Despite growing recognition of the need for a richer characterization of bilingual speakers and of the different contexts of language use, we understand relatively little about the boundary conditions of putative “bilingualism” effects. Here, we review recent findings that demonstrate how variability in the language experiences of bilingual speakers, and also in the ability of bilingual speakers to adapt to the distinct demands of different interactional contexts, impact interactions between language use, language processing, and cognitive control processes generally. Given these findings, our position is that systematic variation in bilingual language experience gives rise to a variety of phenotypes that have different patterns of associations across language processing and cognitive outcomes. The goal of this paper is thus to illustrate how focusing on systematic variation through the identification of bilingual phenotypes can provide crucial insights into a variety of performance patterns, in a manner that has implications for previous and future research.

Dime con quién andas, y te diré quién eres.

Tell me with whom you walk, and I will tell you who you are.

Spanish Proverb

## 1. Introduction

Over the past decade, there has been a marked change in our understanding of bilingual language experience. Whereas past approaches conceptualized variation across samples and/or conditions as deviant or noisy phenomena, recent discoveries point to fundamental interactivity and plasticity in bilingual language learning and processing (Green and Kroll 2019). The emergence of this work has sparked a paradigm shift in the field, resulting in an upsurge of research on individual differences and of comparative studies that seek to exploit variability within and across languages and interactional contexts of language use (for reviews, see de Bruin 2019; Dussias et al. 2019; Fricke et al. 2016; Kroll et al. 2018; Titone and Tiv forthcoming). The changing landscape reflects increased recognition of the complexity of bilingualism as a life experience: bilingualism does not, in itself, result in a particular pattern of response; rather, it is a multidimensional construct that is shaped by individual and contextual factors (Baum and Titone 2014; DeLuca et al. 2020; Luk and Bialystok 2013; Zirnstein et al. 2019). Thus, a key issue is that the differences in trajectories and outcomes of bilingualism are best understood by recognizing the extent of human diversity from an evolutionary perspective (e.g., Henrich et al. 2010; Mason et al. 2015) and by situating sources of individual variance in the sociocultural and linguistic niche within which bilinguals act (Bak 2016; Green 2011; Raviv et al. 2019; Titone and Tiv forthcoming; Wigdorowitz et al. 2020).

Our position is that systematic variation in bilingual language experience gives rise to a variety of *phenotypes*^1^ with different patterns of associations across language processing outcomes. An implication that follows is that a particular association between two variables might be robust for one phenotype, yet absent for others, and so, characterizing speakers in terms of their profile and trajectory through different contexts is essential if we are to understand the limits and boundary conditions of putative bilingualism effects (Green and Abutalebi 2013; Green et al. 2007; Navarro-Torres et al. 2021).

In this paper, we provide an overview of this approach and show how it can be applied to develop an international network for research on diverse bilingual populations using an array of complementary multidisciplinary methods. In a similar spirit to Green and Abutalebi’s (2013) adaptive control hypothesis, we consider three interrelated paths of influence to adaptive change: competition, cooperation, and regulation. We review recent findings that demonstrate how variability in the language experiences of bilingual speakers, and also in the ability of bilingual speakers to adapt to distinct demands of different interactional contexts, impact interactions between language representation, access, and control. Notably, we show that the bilingual language system is dynamic, flexible, and adept at adapting itself to the context of language use in which the speaker is immersed.

In today’s globalized world, individuals are increasingly shifting between distinct environments. In some circumstances, a shift from one interactional context to another results in changes in the relative support for each language (e.g., a Basque-Spanish bilingual living in Andalusia would find little support for the first language (L1)). One question that arises is whether language regulation and language control processes are differentially coordinated for individuals whose interactional circumstances dynamically change. Moreover, bilingualism is pervasive throughout the world, but its manifestation can vary widely among different places, communicative contexts, and individuals (Grosjean 1982, 2013). Bilingual speakers differentially distribute their languages with different people and topics and across everyday settings, such as the classroom/workplace or the home environment (Shiron et al. 2021; Tiv et al. 2020). Some bilinguals typically keep their languages separate; others codeswitch and make use of more than one language opportunistically. We note, too, that interactional contexts differ in the relative intensity or diversity of language use (Gullifer and Titone 2020; Pot et al. 2018; Wigdorowitz et al. 2020). Bilinguals in more variable contexts are presumed to closely monitor the situational context to ensure the appropriateness of their language choices so as to avoid or reduce the interactional cost^2^ that may arise in conversation (Beatty-Martínez et al. 2020b; Green and Abutalebi 2013). Thus, an outstanding question is whether bilinguals who live in more linguistically varied contexts are differentially affected compared to those immersed in relatively more homogeneous environments, where there is a higher degree of certainty with respect to language use and the types of conversational exchanges that take place.

## 2. Competition, Cooperation, and Regulation: Three Paths of Influence to Adaptive Change

### 2.1. Language Competition

Accumulating evidence shows that interactional effects on the trajectories and outcomes of bilingualism are influenced by the ways in which the two languages are engaged. We first contrast an interactional context in which languages are compartmentalized across distinct communicative contexts. Generally, the native language is the predominant language and the second language (L2) is restricted to more exclusive communicative contexts (e.g., at work or with a specific group of people). As the languages are typically highly specialized and differentiated, there is a high interactional cost for mixing languages in conversation. Therefore, individuals who tend to keep their languages separate also tend to have little-to-no codeswitching experience. An important implication for these individuals is that they can use their proven experience at maximizing language competition to reliably distinguish one language from another and predict which language will be used in a given situation.

Indeed, this inference is supported by electrophysiological research demonstrating a modulation of an early frontal positivity (P2), an index of selective attention, in response to an unexpected language switch (e.g., Kuipers and Thierry 2010). In a series of experiments designed to test sensitivity to codeswitches as a function of interactional experience, Beatty-Martínez and Dussias (2017) found that non-codeswitching bilinguals exhibited a larger early frontal positivity when processing a codeswitch relative to a unilingual control. Importantly, these individuals were highly proficient Spanish-English bilinguals living in Granada, Spain and whose linguistic profile and behavioral ecology closely fitted the characterization described above. The switch effect was notably absent when bilinguals processed unilingual translation equivalent sentences in the L1 and L2 separately, further suggesting that the component’s modulation cannot be attributed to differences in L1 versus L2 processing but are due to the selective gating of information flow from one language to the other.^3^ As we will see below, bilinguals’ electrophysiological response to codeswitches depends on the precise form of the gating, namely, whether language control is coordinated competitively (i.e., requiring a narrow attentional focus to exploit one language to the exclusion of another) or cooperatively (i.e., requiring a broad attentional focus to explore both languages opportunistically; see Green and Wei 2014 for theoretical discussion).

Further evidence for the differential engagement of attentional control in bilinguals who use each of their languages in separate communicative contexts has been shown in verbal (Beatty-Martínez 2021; Kuipers and Thierry 2010) and nonverbal (Ooi et al. 2018) auditory domains. Using the elevator counting tasks from the Test of Everyday Attention (Robertson et al. 1994), Ooi et al. (2018) observed that Edinburgh bilinguals who reported using their two languages independently exhibited greater auditory attentional switching abilities when reorienting from one auditory source to another. The interpretation is that bilinguals who avoid codeswitching and compartmentalize their language use across communicative contexts must become adept at reliably adjusting to relevant variations in the input such as a change in language by validating incoming sources of information against their experience-based expectations.

As alluded to previously, interactional contexts in which bilinguals’ languages are used in distinct communicative contexts are characterized by a low degree of language entropy^4^ because the appropriate language is highly predictable. However, language expectations are not always met (e.g., running into your L2-speaking boss at the grocery store). Arguably, such circumstances may trigger a need to reduce between-language interference by reactively suppressing the non-target language to guarantee retrieval in the target language. A plausible conjecture is that such interactional experiences are associated with increased reliance on reactive control processes (i.e., engagement of goal-relevant information on an as-needed basis as a function of changing task demands; Braver 2012) and, conversely, reduced reliance on context monitoring.

Data from behavioral and neuroimaging studies support this assumption. Gullifer et al. (2018) investigated individual differences in resting-state functional connectivity in French-English bilinguals living in Montréal, Canada. The bilinguals examined were all highly proficient in the two languages but varied widely with respect to their measured degree of language entropy within communicative contexts. Relative to bilinguals with more variable interactional experiences, bilinguals with low language entropy exhibited greater reliance on reactive control processes, as measured by the AX continuous performance task (AX-CPT; Ophir et al. 2009), and less connectivity between the anterior cingulate cortex and the putamen, regions previously implicated in monitoring, language switching, and L2 articulatory processing (Abutalebi et al. 2013; Klein et al. 1994). Similarly, Beatty-Martínez et al. (2020b) found that for Spanish-English bilinguals in Granada, greater reliance on reactive control processes in the AX-CPT was associated with better picture naming accuracy in both the L1 and L2. Taken together, the converging evidence reviewed thus far indicates that bilinguals whose interactional experiences center on compartmentalized language use are adept at attentively discriminating one language from another and appear to rely on reactive components of control to manage between-language interference. In the next section, we consider the interactional implications for bilinguals who habitually codeswitch, using their languages freely and interchangeably within different communicative contexts.

### 2.2. Language Cooperation

In codeswitching contexts, where most individuals actively use more than one language, and switching between them is prevalent, bilinguals have the potential to make use of either language on an opportunistic basis to achieve their communicative goals. Decades of sociolinguistic research have documented codeswitching patterns of bilingual speakers resulting in comprehensive corpora of interviews, surveys, and ethnographic research. Quantitative analysis of these data has revealed the systematicity underlying codeswitching tendencies by exemplifying bilinguals’ adherence to community norms over idiosyncratic behaviors (Poplack 1980, 1987; Poplack and Meechan 1998; Torres Cacoullos and Travis 2015, 2018). We have come to see that codeswitching is not random between-language interference but rather serves as an opportunistic strategy^5^ that provides communicative precision (Beatty-Martínez et al. 2020a; Feldman et al. 2021; Xu et al. 2021a, 2021b).

If the implication is that the interactional cost of switching between languages is lower compared to that in contexts in which the two languages are used separately, one could ask whether experience with codeswitching modulates the engagement of language control networks. Particularly strong evidence for this comes from a magnetoencephalographic study on Arabic-English bilinguals investigating language switching in ecologically valid experimental paradigms. Blanco-Elorrieta and Pylkkänen (2017) found that the anterior cingulate and prefrontal cortex, regions implicated when language switching is externally cued (e.g., Abutalebi and Green 2016), showed less involvement during the comprehension of naturalistic codeswitched conversations. Moreover, several other studies have thus far revealed no consistent pattern of association between codeswitching behavior and domain-general cognitive control (Beatty-Martínez et al. 2020b; Hartanto and Yang 2016; Ooi et al. 2018; Pot et al. 2018). Why might this be?

From a theoretical standpoint, because both languages are widely known and routinely used interchangeably, it is not as necessary for bilinguals in codeswitching contexts to continuously monitor the appropriate language and adjust accordingly for each communicative interaction (see Costa et al. 2009 for related evidence on the relation between cognitive control engagement and high monitoring demands). Recent proposals posit that codeswitching contexts involve a cooperative rather than a competitive relation between the two languages and thus offer opportunities for language integration (Calabria et al. 2018; Green and Wei 2014; Green 2019). Therefore, one possibility is that codeswitching creates a context in which bilinguals may adopt an open control mode, in which language membership is minimized and resources from both languages are explored.

To illustrate this point, we return to the findings of Beatty-Martínez and Dussias (2017) introduced in Section 2.1. In addition to the non-codeswitchers from Granada, Beatty-Martínez and Dussias examined a group of Spanish-English bilinguals who were raised in established codeswitching communities in the United States. Contrary to the non-codeswitchers, the codeswitching bilinguals exhibited an N400 modulation (indexing difficulties of semantic integration) in response to infelicitous codeswitches, indicating they were sensitive to codeswitching conventions and community norms (see Adamou and Shen 2017; Beatty-Martínez 2019; Guzzardo Tamargo et al. 2016; Halberstadt 2017 for similar findings). More pertinent to our discussion is that codeswitching bilinguals did not show a modulation of the early frontal positivity when processing a codeswitch relative to a unilingual control. In line with our discussion above, the lack of differentiation between codeswitched and unilingual stimuli at early stages of processing is particularly noteworthy because it suggests that bilinguals’ breadth of selective attention can be broadened to include both language networks. More recently, this finding was followed up in a subsequent study by Kaan et al. (2020), who examined whether bilinguals could dynamically shift between attentional control states depending on the nature of a given conversational exchange. They reported that the early frontal positivity effect was largest when bilinguals were in the presence of a monolingual interlocutor (i.e., where codeswitching was inappropriate), further exemplifying the role of the interactional demands of different contexts in mediating the ways in which bilinguals’ languages are engaged.

At this point, it is useful to distinguish between two interactional experiences that are often conflated in research. While it may be tempting to associate codeswitching with a greater diversity of language experience, we note that codeswitching contexts rely on conventionalized distributional regularities and thus exert relatively uniform interactional demands on language use (Guzzardo Tamargo et al. 2016; Poplack 1987; Poplack et al. 1988; Torres Cacoullos and Travis 2018). Critically, diversity of language use can vary both within and across forms of conversational exchanges. We therefore necessarily distinguish bilinguals’ propensity to engage in codeswitching (i.e., within-speaker language diversity) from their propensity to engage in variable types of conversational exchanges (i.e., betweenspeaker language diversity), which we elaborate on in the next section.

### 2.3. Language Regulation

Thus far, we have characterized interactional contexts of language use with relatively straightforward features: competitive environments where languages are used independently across distinct communicative contexts, and cooperative environments where codeswitching among bilinguals is the norm. Notwithstanding, there are many interactional contexts that involve variable kinds of conversational exchanges, requiring bilinguals to closely monitor and regulate the activation of both languages to suit demands in everyday life. Following the same logic as before, the conjecture is that high-entropy contexts are expected to place greater reliance on proactive control processes (i.e., active engagement and the maintenance of goal-relevant information to execute task demands; Braver 2012) to manage potential between-language interference by keeping the appropriate language active while seeking new contextual cues that may signal a language change (e.g., Pivneva et al. 2014). One strand of evidence relates to research on individual differences in brain-behavior associations in contexts with substantial variability in language diversity, such as Singapore or Montréal. In a series of studies aimed at examining diversity in social language use among Montréal bilinguals, Gullifer and colleagues (Gullifer et al. 2018; Gullifer and Titone 2021) reported that bilinguals with high language entropy showed increased reliance on proactive control in the AX-CPT, as well as greater functional connectivity between the anterior cingulate cortex and the putamen, regions implicated in monitoring and goal maintenance (Abutalebi et al. 2013; see also Li et al. 2021, for corroborative evidence with bilinguals from Singapore).

A second source of evidence comes from research on language use in an L2-immersion context (for reviews see DeLuca et al. 2020; Fricke et al. 2016; Kroll et al. 2018; Kroll et al. 2021; Zirnstein et al. 2019). L2-immersion contexts provide a unique opportunity for examining the dynamic interplay between languages when bilinguals have restricted access to the L1. A considerable body of research has revealed a decline in L1 accessibility with increasing L2 exposure (e.g., Baus et al. 2013; Linck et al. 2009; Whitford and Titone 2012, 2015), suggesting that bilinguals must exert great effort to adjust and regulate co-activation (notably, of the L1 or dominant language) to accommodate to changes in the relative support for each language. There is also evidence that bilingual regulation ability supports proficient language processing by mediating cognitive control recruitment strategies in real time. For example, Zirnstein et al. (2018) examined a group of L2-immersed Mandarin-English bilinguals and found that the bilinguals’ ability to recover from prediction errors during L2 reading was jointly influenced by their L1 regulatory ability and their cognitive control skills. Specifically, increased cognitive control ability related to reduced prediction error costs but only for bilinguals with better L1 regulation. Moreover, a visual world study by Navarro-Torres et al. (2019) found that L2-immersed bilinguals living in Edinburgh proactively disengaged from incorrect interpretations of syntactically ambiguous sentences by relying on early linguistic cues to preempt potential ambiguity.

Similar associations have been observed in language production. Beatty-Martínez et al. (2020b) found that for L2-immersed Spanish-English bilinguals in the United States, greater reliance on proactive control processes in the AX-CPT was associated with better picture-naming accuracy in the L1. Importantly, this pattern of association was absent for Spanish-English bilinguals living in an L1 context (i.e., bilingual groups from Granada and Puerto Rico, whose findings were alluded to in previous sections). The interpretation is that in L2-immersion, bilinguals’ ability to regulate the L1 by proactively monitoring when and when not to use each language can help maintain lexical accessibility in the less-supported L1 (see Zhang et al. forthcoming for corroborating electrophysiological evidence). Taken together, the emerging picture suggests that high-entropy contexts and L2-immersion environments can exert notable consequences for language performance and cognitive control engagement, even in highly proficient bilinguals, by introducing a stronger pressure for regulating coactivation and monitoring the appropriateness of using each language.

## 3. Conclusions

In this paper, we reviewed exciting new findings on how bilingual speakers adapt to the distinct demands of different interactional experiences. This approach leverages the varying experiences across different interactional contexts of language use to identify bilingual phenotypes under different boundary conditions. What is promising about this approach is that it revealed that bilinguals presumed to have been drawn from the same underlying population can differ in significant ways, and even those who might appear to behave similarly can arrive at the same outcome through different routes (Kroll et al. 2021; see Navarro-Torres et al. 2021 for a theoretical discussion on the role of evolutionary and ecological factors in shaping variation in language and cognitive processing).

We emphasize that the identification of bilingual phenotypes is fundamentally a transdisciplinary endeavor. In the last several years, exciting new synergies have emerged between research on bilingualism and other disciplines, such as information theory (Feldman et al. 2021; Gullifer and Titone 2020), network science (Tiv et al. 2020; Titone and Tiv forthcoming; Xu et al. 2021a, 2021b), and usage-based approaches (Beatty-Martínez et al. 2018; Navarro-Torres et al. 2021). In this respect, a focus on multi-lab collaborations aimed at leveraging different interactional experiences offers promising prospects for embedding such work in a more comprehensive view of adaptive change (Kroll et al. 2018; Leivada et al. 2020). As a field, we are in the early stages of understanding the precise aspects of bilingual experience that give rise to different trajectories and outcomes. Notwithstanding, we have attempted to show that by providing a rich characterization of bilingual speakers in terms of their habits of language use and in relation to their interactional context, we can more effectively extract signals from noise.

## Footnotes

1 A phenotype is simply the characterization of the relevant features (e.g., traits, skills, sociocultural practices, etc.) that explain the adaptive interplay between the brain and the environment for a population of speakers. The identification and characterization of which features are relevant for a given population of speakers is critical for research on bilingualism (see Navarro-Torres et al. 2021 for further illustration).

2 Consider a French-English bilingual living in Montréal who reports using French primarily at home, English in the workplace, and codeswitching regularly among friends. Language control processes must adapt to the demands of different contexts of language use to avoid the cost that may arise from not doing so (see Green and Abutalebi 2013 for further discussion).

3 We follow Green and Wei (2014) in the use of the term ‘gate’ to emphasize how language control states may either restrict or allow access to information from either one or more language(s) subject to the appropriateness of codeswitching.

4 Briefly, language entropy is a metric for calculating the relative balance or diversity of language use and/or exposure within and across communicative contexts. Lower entropy values indicate increased certainty about when a particular language will occur at a given time, whereas higher values indicate the opposite (see Gullifer and Titone 2018 for a fully-documented R package).

5 Recent findings have shown that for codeswitching bilinguals, the likelihood of switching between languages increases when the word in the other language is more accessible than the equivalent word in the current language (Xu et al. 2021a, 2021b), under conditions of greater lexical diversity (Feldman et al. 2021), and when words or structures in the other language provide greater discriminatory efficiency (Beatty-Martínez et al. 2020a). What this suggests is that codeswitching offers a unique and flexible feature of bilingualism through which resources from both languages are recruited to provide an alternative means to convey a communicative intention, with implications for language control and speech planning.
